# Patient‐reported symptom burden in routine oncology care: Examining racial and ethnic disparities

**DOI:** 10.1002/cnr2.1478

**Published:** 2021-06-24

**Authors:** Hailey W. Bulls, Pi‐Hua Chang, Naomi C. Brownstein, Jun‐Min Zhou, Aasha I. Hoogland, Brian D. Gonzalez, Peter Johnstone, Heather S. L. Jim

**Affiliations:** ^1^ Department of Health Outcomes and Behavior Moffitt Cancer Center Tampa Florida USA; ^2^ Division of General Internal Medicine, Department of Medicine University of Pittsburgh Pittsburgh Pennsylvania USA; ^3^ Department of Nursing Taichung Veterans General Hospital Taichung Taiwan, ROC; ^4^ School of Nursing College of Medicine, National Taiwan University Taipei Taiwan, ROC; ^5^ Department of Biostatistics and Bioinformatics Moffitt Cancer Center Tampa Florida USA; ^6^ Department of Radiation Oncology Moffitt Cancer Center Tampa Florida USA

**Keywords:** ethnic disparities, oncology, pati, ent‐reported outcomesracial disparitiessymptom assessment

## Abstract

**Background:**

Racial and ethnic disparities are well‐documented in cancer outcomes such as disease progression and survival, but less is known regarding potential disparities in symptom burden.

**Aims:**

The goal of this retrospective study was to examine differences in symptom burden by race and ethnicity in a large sample of cancer patients. We hypothesized that racial and ethnic minority patients would report greater symptom burden than non‐Hispanic and White patients.

**Methods and results:**

A total of 5798 cancer patients completed the Edmonton Symptom Assessment Scale—revised (ESAS‐r‐CSS) at least once as part of clinical care. Two indicators of symptom burden were evaluated: (1) total ESAS‐r‐CSS score (i.e., overall symptom burden) and (2) number of severe symptoms (i.e., severe symptomatology). For patients completing the ESAS‐r‐CSS on multiple occasions, the highest score for each indicator was used. Zero‐inflated negative binomial regression analyses were conducted, adjusting for other sociodemographic and clinical characteristics. Symptomology varied across race. Patients who self‐identified as Black reported higher symptom burden (*p* = .016) and were more likely to report severe symptoms (*p* < .001) than self‐identified White patients. Patients with “other” race were also more likely to report severe symptoms than White patients (*p* = .032), but reported similar total symptom burden (*p* = .315). Asian and Hispanic patients did not differ from White or non‐Hispanic patients on symptom burden (*p*s > .05).

**Conclusion:**

This study describes racial disparities in patient‐reported symptom burden during routine oncology care, primarily observed in Black patients. Clinic‐based electronic symptom monitoring may be useful to detect high symptom burden, particularly in patients who self‐identify their race as Black or other. Future research is needed to reduce symptom burden in racially diverse cancer populations.

## 1 INTRODUCTION

Racial and ethnic disparities in cancer outcomes are well‐documented and evident at every stage of the cancer continuum, from prevention through active treatment and into survivorship.^1^, ^2^, ^3^ Specific disparities related to race include lower cancer screening rates, higher incidence of certain cancers (e.g., multiple myeloma, colorectal, lung, cervical, and triple‐negative breast cancer), increased perioperative mortality, and increased cancer‐specific and overall mortality.^3^, ^4^, ^5^, ^6^ Reduction of health disparities is increasingly recognized as an important national goal.^7^, ^8^, ^9^ One potential disparity that has received less attention is symptom burden.

Literature directly examining symptom burden among racial and ethnic groups is limited, typically focusing on single symptoms within specific cancer diagnoses in Black versus White patients. For example, Black or Hispanic women with breast cancer are more likely to report pain, skin irritations, and limitations in physical function when compared to those who are non‐Hispanic and White.^10^, ^11^, ^12^, ^13^, ^14^ Racial and ethnic disparities in symptom management have also been documented. Black patients report high levels of unmet needs in symptom management.^15^, ^16^, ^17^ One study found that US‐born Black patients and foreign‐born Asian and Hispanic patients were up to 10.9% more likely to perceive an unmet supportive care need than White, US‐born patients.^15^ Further investigation is important to fully identify racial and ethnic disparities in symptom burden because under‐ or un‐treated symptoms can lead to poor quality of life, higher rates of emergency department use, treatment non‐compliance, end‐of‐life hospital admissions, and worse clinical outcomes.^15^, ^18^, ^19^, ^20^ One way to evaluate symptom burden is through electronic clinic‐based symptom assessments as part of routine clinical care.

Patient‐reported outcomes (PROs) offer distinct information from provider‐assessed adverse event reporting. For example, in a 2010 study of 1833 patient‐health care provider dyads, providers significantly underestimated the presence of severe pain, fatigue, generalized weakness, anorexia, depression, constipation, poor sleep, dyspnea, nausea, vomiting, and diarrhea.^21^ Another study comparing PROs with physician‐assessed adverse events found that physicians under‐reported severe treatment‐related toxicities by up to 50%; under‐reporting symptoms of any severity ranged up to 74%.^22^ Conversely, recent studies indicate that clinic‐based PRO assessment and symptom management results in better outcomes including improved patient‐clinician communication, clinician awareness of patient symptoms, treatment decision making, healthcare utilization, patient satisfaction, quality of life, and survival.^23^, ^24^, ^25^, ^26^, ^27^, ^28^, ^29^

The goal of the current, retrospective study was to examine potential racial and ethnic disparities in patient‐reported symptom burden in adult oncology patients, controlling for other sociodemographic and clinical characteristics. It was hypothesized that patients self‐identifying as a member of a racial or ethnic minority group (i.e., Black, Hispanic) would report higher total symptom burden and more severe symptoms compared to non‐Hispanic and White patients.

## 2 METHODS

### 2.1 Participants and procedures

Patients presenting to the Moffitt Radiation Oncology or Supportive Care Medicine clinics completed the Edmonton Symptom Assessment Scale—revised (ESAS‐r‐CSS)^30^ as part of routine clinical care. The strength of using a clinical dataset is that there is no recruitment bias. Questionnaires were time‐ and date‐stamped upon completion. Patients were included in analyses if they were 18 years of age or older and had completed at least one symptom assessment. The study was approved by the Advarra Institutional Review Board.

### 2.2 Measures

*Demographic and clinical data*: Demographic and clinical characteristics were extracted from Moffitt internal databases. Variables included date of birth, sex, self‐identified race, self‐identified ethnicity, marital status, primary cancer site, and cancer status (active disease vs. no active disease). Race was categorized as White, Black, Asian, and other. The “other” category comprised a combination of racial groups with small sample sizes (American Indian, Aleutian, or Eskimo; More than 1 race; Native Hawaiian or Other Pacific Islander; and Other). Ethnicity was categorized as Hispanic and non‐Hispanic.

*Symptom burden*: A modified version of the ESAS‐r‐CSS^30^ was used to evaluate symptoms. The ESAS‐r‐CSS is a 12‐item questionnaire that assesses the presence and severity of 12 core symptoms, including pain, tiredness, drowsiness, nausea, lack of appetite, shortness of breath, depression, anxiety, overall well‐being, spiritual well‐being, constipation, and difficulty sleeping. Patients rate each symptom on an 11‐point Likert scale (i.e., 0 = none and 10 = worst possible) based on their symptoms at the time of questionnaire completion. Items were summed to create a total score (0–120). Higher scores indicate greater symptom burden. Individual symptoms were considered severe if they are rated 7 or greater.^31^ The ESAS‐r‐CSS was administered on paper forms from January 2015 to January 2017 and via an electronic tablet thereafter.^31^ All forms completed between January 2015 and June 2018 were included in analyses.

### 2.3 Statistical analyses

Because the combined effect of several mild or moderate symptoms may be as burdensome as a severe symptom, symptom burden was assessed by two derived variables: (1) ratings of all symptoms were summed to provide a total symptom burden score and (2) the number of symptoms rated as severe was summed to separately to capture severe symptomatology. For patients with data from multiple clinic visits, the highest score for each variable (total symptom burden and number of severe symptoms) was used. Scores for total symptom burden and number of severe symptoms could have been reported on the same clinic visit or different clinic visits. For example, a patient could have their highest total symptom burden recorded at a different time than their highest count of severe symptoms.

Patients' demographic and clinical characteristics were summarized using descriptive statistics, and the distributions of the outcome variables were inspected visually via histograms (not shown). Zero‐inflated negative binomial (ZINB) models were conducted separately for both outcome variables, controlling for sociodemographic and clinical characteristics. ZINB models are appropriate for modeling count variables with high numbers of zeros and over‐dispersion. In this case, ZINB analyses account for the relatively high numbers of patients reporting no symptom burden and the fact that the average and variance differed for each symptom burden measure. ZINB analyses consist of two components, a zero‐inflation component to predict patients that have zero burden, and a negative binomial model to measure the burden score for patients not already predicted to have zero burden. First, multivariable logistic regression models were fit to identify relevant variables for inclusion in the ZINB models as predictors of zero symptom burden. Variable selection for the logistic regression models was evaluated by comparing the results of backward selection (using a criterion of 0.05 for removal of a variable) and best subset selection.^32^ Second, ZINB models were conducted consisting of models for probability of zero burden (the zero‐inflation component) and symptom burden score (the negative binomial component). Variables selected in the first step for inclusion in the logistic regression model were included as predictors in the zero‐inflation component of the ZINB model for having a symptom burden value of zero. With the zero‐inflation parameters held fixed, backward selection with a criterion of 0.05 was utilized to select the variables for inclusion in the negative binomial model for symptom burden. However, for the negative binomial components, race and ethnicity were automatically considered as predictors due to the focus of the research question (i.e., race and ethnicity were in the final ZINB models for symptom burden for purpose of quantifying their effect sizes whether or not the variables were statistically significant). Finally, the appropriateness of the ZINB model was compared with standard negative binomial regression using the Vuong test.^33^ Statistical analyses were performed using SAS 9.4 (Cary, NC).

## 3 RESULTS

### 3.1 Patient characteristics

Sociodemographic and clinical characteristics of the 5798 patients are shown in Table 1. The majority of the sample was non‐Hispanic (91%), White (86%), and male (54%). Patients ranged in age from 18 to 97, with an average age of 64 (SD = 13). The three most common cancer diagnoses were lung (18%), breast (18%), and male genitourinary (16%). Slightly more than half of the sample had active cancer at the time of assessment (53%). A majority of those with known marital status were married (69%). However, marital status was missing for 16% of patients. Due to this high proportion of missing data, marital status was not considered for inclusion in the ZINB models.

**TABLE 1:** Participant Characteristics, *N* = 5798

	Sum‐Max	Severe‐Max
Age: *M* (SD)	64.12 (12.51)	64.09 (12.5)
Aged 65 or older: *N* (%)	2963 (51.1)	2957 (51.0)
Race: *N* (%)		
White	4859 (85.9)	4859 (85.9)
Black/African‐American	419 (7.4)	419 (7.4)
Asian	95 (1.7)	95 (1.7)
Other	284 (5.0)	284 (5.0)
Missing	141	141
Ethnicity: *N* (%)		
Hispanic	492 (8.7)	492 (8.7)
Non‐Hispanic	5162 (91.3)	5162 (91.3)
Missing	144	144
Gender: *N* (%)		
Male	3133 (54.0)	3133 (54.0)
Female	2664 (46.0)	2665 (46.0)
Missing	1	0
Marital status: *N* (%)		
Married	3347 (68.6)	3347 (68.6)
Single	663 (13.6)	663 (13.6)
Divorced	521 (10.7)	521 (10.7)
Widowed	293 (6.0)	293 (6.0)
Separated	30 (0.6)	30 (0.6)
Domestic partner	25 (0.5)	25 (0.5)
Missing	919	919
Primary cancer site: *N* (%)		
Lung	1064 (18.4)	1064 (18.4)
Breast	1012 (17.5)	1011 (17.5)
Male genitalia	941 (16.3)	942 (16.3)
Head and neck	695 (12.0)	696 (12.0)
Gastrointestinal	417 (7.2)	417 (7.2)
Skin	392 (6.8)	391 (6.8)
Hematological	318 (5.5)	318 (5.5)
Gynecologic	266 (4.6)	266 (4.6)
Sarcoma	218 (3.8)	218 (3.8)
Neurological	191 (3.3)	191 (3.3)
Genitourinary	154 (2.7)	154 (2.7)
Endocrine	80 (1.4)	80 (1.4)
Bone	35 (0.6)	35 (0.6)
Missing	15	15
Cancer disease status: *N* (%)		
No active disease	2555 (47.1)	2555 (47.1)
Active	2872 (52.9)	2873 (52.9)
Missing	370	371
ESAS Sum‐Max: *M* (SD), range	31.2 (24.5), 0–116	
ESAS Sum‐Max = 0: *N* (%)	356 (6.2)	
ESAS Severe‐Max: *M* (SD), range		2.0 (2.5), 0–12
ESAS Severe‐Max = 0: *N* (%)		2361 (42.0)

*Note*: Percentages calculated from available data.

Patients completed a total of 19 670 individual surveys. Fewer than 3% of patients were missing any ESAS information. The median and range of surveys contributed by each patient was 2 (1–29). The highest overall symptom burden and highest severe symptom burden were retained for each of the 5798 patients. Patients' average worst overall symptom burden score was 31.2 (SD = 24.5) out of a possible score of 120. Responses ranged from 0 to 116, with a median of 24. An overall worst symptom burden of zero was reported by 356 patients (6.2%). Participants' average highest number of severe symptoms was 2.0 (SD = 2.5) out of a possible score of 12. Responses ranged from no severe symptoms to all 12 symptoms rated as severe, with a median of 1. No severe symptoms were reported by 2361 patients (42.0%).

### 3.2 Modeling overall symptom burden

The first step of the ZINB models was to identify predictors of zero symptom burden using multivariable logistic regression models. Results of the backward selection logistic regression analysis indicated that being male, not having active disease, and location of the primary cancer site were variables associated with higher odds of having a symptom burden of zero (Table S1). This choice of three variables was confirmed by best subset selection. However, inclusion of the primary cancer site resulted in an infinite parameter estimate and was problematic in the logistic regression model. Similarly, if included, primary cancer site would subsequently impair the zero‐inflation portion of the ZINB model. Thus, while the other predictors (disease status and gender) were retained, primary cancer site was not included in the portion of overall symptom burden ZINB analyses that would predict zero total scores. Prediction of zero symptom burden was not improved by including race or ethnicity. A detailed table is included in the supplementary material.

The second step was to fit the complete ZINB models, holding the zero‐inflation predictors (gender and active cancer disease status) fixed based on the modeling described in the previous paragraph. In addition to race and ethnicity, which were included in the negative binomial component of the model regardless of their effect sizes and *p*‐values, variables selected into the final negative binomial model were age, gender, primary cancer site, and cancer disease status. Results of this model are shown in Tables 2 and 3, for the negative binomial and zero‐inflation components, respectively. According to the Vuong test,^33^ the zero‐inflated negative binomial model was preferable to a standard negative binomial model without accounting for the extra patients with zero symptom burden (*p* < .0001). In the zero‐inflation component (Table 3), consistent with the logistic regression model, females had 57% lower odds than males and of having a zero total symptom score. The overall symptom burden is then reported in the negative binomial component (Table 2) for patients not already predicted to have zero burden. Race was associated with overall symptom burden after controlling for other variables (*p* = .017) for patients predicted to have nonzero total burden. Specifically, the expected score for Black patients predicted to have nonzero burden is 11% higher than the expected score for Whites with all other variables held equal. The expected total score for Asian patients with nonzero burden is 15% lower than the corresponding expected score for Whites. Patients with “other” race reported total symptom burden scores reasonably similar to White patients (estimated ratio of 1.06 [95% CI: 0.95, 1.19]; *p* = .315). There were no meaningful differences in overall symptom burden between Hispanic and non‐Hispanic patients (*p* = .92). Younger age, having active disease, primary cancer site, and female gender were associated with higher reported symptom burden.

**TABLE 2:** Zero‐inflated negative binomial regression assessing racial and ethnic differences in the maximum total symptom burden

Variable	Estimate	Ratio (95% CI)	*p*‐Value (level)	*p*‐Value (overall)
Race				**.0171**
Black/African‐American (*n* = 389)	0.105	1.11 (1.02–1.21)	**.0163**	
Asian (*n* = 84)	−0.158	0.85 (0.72–1.02)	.0748	
Other (*n* = 253)	0.059	1.06 (0.95–1.19)	.3150	
White (*n* = 4446) (ref)				
Ethnicity				.9195
Hispanic (*n* = 444)	−0.005	1.00 (0.91–1.09)		
Non‐Hispanic (*n* = 4728) (ref)				
Age (*n* = 5172)	−0.008	0.992 (0.991–0.994)	**<.0001**	**<.0001**
Gender				
Female (*n* = 2412)	0.112	1.12 (1.06–1.19)	**.0002**	**.0002**
Male (*n* = 2760) (ref)				
Primary cancer site				**<.0001**
Lung (*n* = 950)	0.220	1.25 (1.13–1.38)	**<.0001**	
Breast (*n* = 932)	−0.072	0.93 (0.84–1.04)	.1886	
Male genitalia (*n* = 818)	−0.252	0.78 (0.70–0.86)	**<.0001**	
Head and neck (*n* = 603)	0.132	1.14 (1.03–1.27)	**.0150**	
Gastrointestinal (*n* = 375)	0.232	1.26 (1.12–1.42)	**<.0001**	
Hematological (*n* = 288)	0.241	1.27 (1.12–1.44)	**.0002**	
Gynecologic (*n* = 245)	0.217	1.24 (1.08–1.42)	**.0019**	
Sarcoma (*n* = 196)	0.141	1.15 (1.00–1.33)	.0503	
Neurological (*n* = 162)	−0.041	0.96 (0.83–1.12)	.5993	
Genitourinary (*n* = 139)	0.246	1.28 (1.09–1.50)	**.0022**	
Endocrine (*n* = 71)	0.169	1.18 (0.97–1.45)	.1010	
Bone (*n* = 28)	0.137	1.15 (0.85–1.55)	.3739	
Skin (*n* = 365) (ref)				
Cancer disease status				
Not active (*n* = 2416)	−0.255	0.77 (0.74–0.81)	**<.0001**	**<.0001**
Active (*n* = 2756) (ref)				

*Note*: In general, larger (positive) estimates indicate greater expected total symptom burden scores. Ratios for categorical variables report the expected total symptom burden among those not already predicted to have zero burden for the comparison group divided by the expected total symptom burden for the reference group. For age, the ratio reports the expected multiplicative change in total symptom burden associated with each additional year of age.

*Note*: For categorical covariates, estimates correspond to the expected change in the logarithm of the symptom score for each comparison group compared to the logarithm of the symptom score for the reference group (e.g., Black/African/American vs. White). Positive estimates (and ratios exceeding one) correspond to higher symptom scores for the comparison group, while negative estimates (and ratios below one) correspond to higher symptom scores for the reference group. For continuous covariates (e.g., age), estimates report the expected change in logarithm of the symptom score per unit change in the covariate.

*Note*: All bolded values are statistically significant; see table for specific *p‐*values.

**TABLE 3:** Analysis of maximum likelihood zero inflation parameter estimates in the maximum total symptom burden

Variable	Estimate	OR (95% CI)	*p*‐Value (level)	*p*‐Value (overall)
Gender				**<.0001**
Female (*n* = 2412)	−0.855	0.43 (0.32–0.56)	**<.0001**	
Male (*n* = 2760) (ref)				
Cancer disease status				
Not active (*n* = 2416)	0.536	1.71 (1.32–2.21)	**<.0001**	
Active (*n* = 2756) (ref)				

*Note*: Estimates correspond to the expected change in log‐odds of zero symptom score. Larger (positive) estimates indicate greater odds of zero score. Odds ratios are equal to the odds of having zero total symptom burden in the comparison group divided by the odds of having zero total symptom burden in the reference group.

*Note*: For categorical covariates, estimates correspond to the expected change in the logarithm of the symptom score for each comparison group compared to the logarithm of the symptom score for the reference group (e.g., Black/African/American vs. White). Positive estimates (and ratios exceeding one) correspond to higher symptom scores for the comparison group, while negative estimates (and ratios below one) correspond to higher symptom scores for the reference group. For continuous covariates (e.g., age), estimates report the expected change in logarithm of the symptom score per unit change in the covariate.

*Note*: All bolded values are statistically significant; see table for specific *p‐*values.

### 3.3 Modeling severe symptomatology

Analyses were conducted with the same procedures described above to identify predictors of zero severe symptoms using multivariable logistic regression models. Results of the backward selection logistic‐regression analysis indicated that older age (*p* = .0364), male sex (*p* = . 0311), absence of active disease (*p* < .0001), and primary cancer site (*p* < .0001) were associated with the probability of no severe symptoms (Table S2). No infinite parameters were observed in this logistic regression analysis. Although marital status was also associated with lower probability of zero severe symptoms, we elected not to carry it forward into the ZINB model because of the sizable number of participants with unknown marital status. The remaining four variables (age, sex, active disease, and primary cancer site) were retained in the portion of the ZINB analyses that predict a score of zero.

ZINB models were then created to examine associations among severe symptoms, race, ethnicity, and other important patient factors chosen by backward selection. Results of this model are shown in Tables 4 and 5. Similar to the findings for total symptom burden, neither race nor ethnicity were associated with the probability of having zero severe symptoms after controlling for other predictors. While the logistic regression model parameters for primary cancer site were stable, the zero‐inflation component resembled the stability concern for primary cancer site in the model for total symptom score. In Table 5, the parameter for bone cancer has a wide confidence interval, likely due to the small sample of patients with bone cancer. Paralleling the findings for total symptom burden, the zero‐inflated negative binomial model for severe symptom burden provided sufficiently more information compared to a model without zero inflation to justify the use of the zero‐inflated model (*p* < .0001).

**TABLE 4:** Zero‐inflated negative binomial regression assessing racial and ethnic differences in the maximum number of severe symptoms

Variable	Estimate	Ratio (95% CI)	*p*‐Value (level)	*p*‐Value (overall)
Race				**.0002**
Black/African‐American (*n* = 397)	0.214	1.24 (1.10–1.40)	**.0006**	
Asian (*n* = 84)	−0.236	0.79 (0.61–1.03)	.0803	
Other (*n* = 261)	0.186	1.20 (1.02–1.43)	**.0324**	
White (*n* = 4514) (ref)				
Ethnicity				.4268
Hispanic (*n* = 454)	0.052	1.05 (0.93–1.20)	.4268	
Non‐Hispanic (*n* = 4802) (ref)				
Age (*n* = 5256)	−0.008	0.993 (0.990–0.995)	**<.0001**	**<.0001**
Gender				**<.0001**
Female (*n* = 2450)	0.183	1.20 (1.10–1.31)	**<.0001**	
Male *n* = 2806) (ref)				
Primary cancer site				**.0002**
Lung (*n* = 973)	0.141	1.15 (0.98–1.36)	.0941	
Breast (*n* = 948)	−0.138	0.87 (0.73–1.04)	.1313	
Male genitalia (*n* = 827)	−0.176	0.84 (0.69–1.02)	.0735	
Head and neck (*n* = 614)	0.104	1.11 (0.93–1.33)	.2567	
Gastrointestinal (*n* = 380)	0.141	1.15 (0.95–1.39)	.1396	
Hematological (*n* = 291)	0.113	1.12 (0.92–1.36)	.2564	
Gynecologic (*n* = 246)	0.173	1.19 (0.97–1.46)	.1025	
Sarcoma (*n* = 199)	0.038	1.04 (0.83–1.30)	.7370	
Neurological (*n* = 164)	−0.128	0.88 (0.69–1.13)	.3137	
Genitourinary (*n* = 140)	0.119	1.13 (0.88–1.44)	.3465	
Endocrine (*n* = 72)	0.208	1.23 (0.91–1.67)	.1778	
Bone (*n* = 28)	−0.292	0.75 (0.46–1.21)	.2327	
Skin (*n* = 374) (ref)				
Cancer disease status				**<.0001**
Not active (*n* = 2457)	−0.224	0.80 (0.74–0.86)	**<.0001**	
Active (*n* = 2799) (ref)				

*Note*: In general, larger (positive) estimates and odds ratios exceeding unity indicate greater expected total symptom burden scores. Ratios for categorical variables report the expected number of severe symptoms among those not already predicted to have zero severe symptoms for the comparison group divided by the expected number of severe symptoms for the reference group. For age, the logarithm of the ratio reports the expected change in number of severe symptoms associated with an additional year of age.

*Note*: For categorical covariates, estimates correspond to the expected change in the logarithm of the symptom score for each comparison group compared to the logarithm of the symptom score for the reference group (e.g., Black/African/American vs. White). Positive estimates (and ratios exceeding one) correspond to higher symptom scores for the comparison group, while negative estimates (and ratios below one) correspond to higher symptom scores for the reference group. For continuous covariates (e.g., age), estimates report the expected change in logarithm of the symptom score per unit change in the covariate.

*Note*: All bolded values are statistically significant; see table for specific *p‐*values.

**TABLE 5:** Analysis of maximum likelihood zero inflation parameter estimates in the maximum number of severe symptoms

Variable	Estimate	OR (95% CI)	*p*‐Value (level)	*p*‐Value (overall)
Age (*n* = 5256)	0.008	1.01 (1.001–1.016)	**.0369**	**.0364**
Gender				**.0311**
Female (*n* = 2450)	−0.029	0.75 (0.57–0.98)	**.0342**	
Male (*n* = 2806) (ref)				
Primary cancer site				**<.0001**
Lung (*n* = 973)	−0.768	0.46 (0.31–0.69)	**.0001**	
Breast (*n* = 948)	−0.067	0.94 (0.62–1.41)	.7492	
Male genitalia (*n* = 827)	0.432	1.54 (1.06–2.24)	**.0238**	
Head and neck (*n* = 614)	−0.312	0.73 (0.50–1.08)	.1173	
Gastrointestinal (*n* = 380)	−0.888	0.41 (0.25–0.69)	**.0008**	
Hematological (*n* = 291)	−0.980	0.38 (0.20–0.69)	**.0015**	
Gynecologic (*n* = 246)	−0.595	0.55 (0.31–0.97)	**.0390**	
Sarcoma (*n* = 199)	−0.917	0.40 (0.21–0.77)	**.0061**	
Neurological (*n* = 164)	−0.194	0.82 (0.44–1.55)	.5484	
Genitourinary (*n* = 140)	−0.723	0.49 (0.24–0.98)	**.0450**	
Endocrine (*n* = 72)	−0.580	0.56 (0.24–1.28)	.1706	
Bone (*n* = 28)	−3.285	0.04 (0.00–52 076)	.6490	
Skin (*n* = 374) (ref)				
Cancer disease status				**<.0001**
Not active (*n* = 2457)	0.641	1.90 (1.57–2.30)	**<.0001**	
Active (*n* = 2799) (ref)				

*Note*: Estimates correspond to the expected change in log‐odds of severe symptoms. Larger (positive) estimates indicate greater expected odds of reporting zero severe symptoms. Odds ratios are equal to the odds of having zero severe symptoms in the comparison group divided by the odds of having zero total symptom burden in the reference group.

*Note*: For categorical covariates, estimates correspond to the expected change in the logarithm of the symptom score for each comparison group compared to the logarithm of the symptom score for the reference group (e.g., Black/African/American vs. White). Positive estimates (and ratios exceeding one) correspond to higher symptom scores for the comparison group, while negative estimates (and ratios below one) correspond to higher symptom scores for the reference group. For continuous covariates (e.g., age), estimates report the expected change in logarithm of the symptom score per unit change in the covariate.

*Note*: All bolded values are statistically significant; see table for specific *p‐*values.

The negative binomial component of the ZINB model facilitates an analysis of the expected number of severe symptoms for patients predicted to have at least one severe symptom. In addition to race and ethnicity, the final negative binomial model included age, sex, primary cancer site, and cancer disease status. Results of the ZINB regression model indicated that race was associated with the count of severe symptoms (*p* = .0002). Specifically, the expected number of severe symptoms for patients predicted to have at least one severe symptom was 24% higher for Blacks, 21% lower for Asian patients, and 20% higher for those of “other” race compared to White patients. No statistically detectable differences in severe symptoms were observed between Hispanic and non‐Hispanic patients (*p* = .43). Females predicted to have nonzero severe burden reported 20% higher expected counts of severe symptoms than males (*p* < .0001). Patients without active disease reported 20% lower expected counts of severe symptoms than did patients with active disease (*p* < .0001).

## 4 DISCUSSION

The goal of this study was to examine potential racial and ethnic disparities in patient‐reported symptom burden and severe symptomatology in cancer patients. We hypothesized that patients self‐identifying as a member of a racial or ethnic minority group (i.e., Black, Hispanic, Asian) would report higher total symptom burden and more severe symptoms compared to non‐Hispanic and White patients. Our hypotheses were partially supported, as analyses revealed disparities in higher overall symptom burden and more severe symptoms reported by Black patients as compared to their White counterparts. These results suggest that there is an unmet need for symptom management in patients who self‐identify as Black, and that supportive care interventions should be explored in order to reduce the high level of severe symptoms and impact of symptom burden in this patient group.

We anticipated that Hispanic patients would report worse symptom burden and more severe symptoms than non‐Hispanic patients, in part due to previous findings that Hispanic cancer patients are at risk of higher symptom burden,^34^ worse psychological distress,^35^ and worse quality of life.^36^ In our study, no differences were observed in either overall symptom burden or number of severe symptoms between Hispanic and non‐Hispanic patients. This finding could be attributed to the relatively small percentage of Hispanic patients in this sample (8%) compared to the general population (18%),^37^ which may itself be in part due to the fact that a Spanish‐language version for the ESAS‐r‐CSS has yet to be deployed in the study clinics. Asian patients reported that their symptoms were marginally less burdensome and severe than White patients. Finally, patients with “other” race also reported more severe symptoms than White patients, though their overall symptom burden was similar.

The multilevel contextual model^38^, ^39^ may be helpful in future studies to identify causes for the cancer health disparities observed in this study. Several potential reasons for these disparities have been posited in previous research, including factors at the individual level (e.g., distress, genetic factors, diet) and the societal level (e.g., socioeconomic status, cultural factors).^4^ An important risk factor for health disparities at the healthcare system level is racially discordant interactions (e.g., a Black patient treated by a White clinician).^40^ Prior research indicates that racially discordant interactions (typically where the patient is a racial minority and the physician is White) are common and perceived by the patient as less positive and productive.^41^ Racially discordant appointments are also shorter in length, and the discussion is typically more physician‐dominated and less patient‐centered.^42^ These factors likely create a barrier in patients' comfort reporting their symptoms to their provider, which may contribute to disparities in effective symptom management for racial minorities. This healthcare system‐level risk factor may be particularly important because it may be more easily modified than some individual‐ and system‐level factors. While research suggests that minority patients may have a better experience when treated by racially concordant providers,^43^ only 2.3% of oncologists self‐identify as Black or African American and 5.8% as Hispanic,^44^ suggesting that efforts are needed to increase representation of minorities in clinical settings. In the meantime, interventions are needed to reduce symptom burden regardless of race and ethnicity, but particularly in Black patients.

Regardless of etiology, one intervention that may address racial disparities in symptom burden is implementation of PROs for all patients as part of routine clinical care. Implementation of electronic clinic‐based PROs results in more patient–clinician communication, better clinician awareness of patient symptoms, better treatment decision making, improved healthcare utilization, increased patient satisfaction, improved quality of life, and better clinical outcomes.^23^, ^24^, ^25^, ^26^, ^27^, ^28^, ^29^, ^45^ Electronic PROs are also feasible to implement in the clinic, with relatively little burden for patient and provider.^46^ For example, Moffitt Cancer Center integrates electronic administration of the ESAS‐r‐CSS into clinical care for all patients presenting to the Radiation Oncology or Supportive Care Medicine clinics, with real‐time integration into the electronic medical record (EMR). Severe symptoms are highlighted in the EMR, cueing providers to follow up in the clinic visit to determine whether intervention is warranted (e.g., medication change, referral to supportive care). Future research should assess whether implementing such a program results in improved outcomes for cancer patients of racial minority backgrounds.

Strengths of this study include a large, heterogeneous sample of cancer patients; multiple measures of symptomatology (e.g., overall symptom burden, number of severe symptoms); routine collection of data as part of clinical care that includes all patients; and an integrated electronic medical record system that allowed ESAS‐r‐CSS assessments to be linked with sociodemographic and clinical characteristics. Moreover, there were relatively large numbers of Black and Hispanic participants (400+ each) included in the analysis. Limitations include heterogeneous times of assessment relative to disease and treatment events and lack of data on cancer treatments received. Analyses presented in this paper use the presence of active disease as a control variable. Given the heterogeneity of the cancer types included, differing staging systems (e.g., TNM vs. others), and variability in staging documentation (e.g., clinical vs. pathological staging), we were unable to include a standardized staging variable in our analyses. Future studies focused on specific disease types should explore the role of disease stage in patient‐reported symptom burden and potential racial disparities. Additionally, a number of potential sociodemographic variables were unavailable in the current dataset, such as data about the patient's neighborhood, community/family support (besides marital status), and other social determinants of health. Future research should build on this study to examine the impact of other sociodemographic factors that may influence disparities in symptom burden. Nevertheless, the goal of the current study was to explore the presence of racial and ethnic differences in symptoms among oncology patients in clinical practice, which provides a basis for continued research promoting a comprehensive understanding of racial disparities in patient‐reported symptoms. Additionally, symptom data were not collected from patients presenting to clinics other than Radiation Oncology or Supportive Care Medicine.

Despite limitations, this study contributes to the growing body of literature describing racial disparities in cancer outcomes, including patient‐reported symptom burden during routine oncology care. Results offer support for the use of clinic‐based electronic symptom monitoring to identify high symptom burden in oncology populations and provide a foundation for continued work evaluating clinical and social contributors to racial disparities in patient‐reported symptomatology. Future research is needed to determine whether use of electronic, clinic‐based PROs improves patient/physician communication, cues providers to address potentially unmet supportive care needs, and results in reduced disparities in symptom burden.

### AKNOWLEDGEMENTS

This study was supported by the U.S. National Cancer Institute grants R25 CA090314 (PI: Brandon), K01 CA211789 (PI: Gonzalez), R01 CA214647 (PI: Jim), R01 CA219389 (PI: Jim), P30 CA076292 (PI: Cleveland) and the Taichung Veterans General Hospital and Veterans Affairs Council, Taiwan, R.O.C. (No. 2018Y‐77). Additional support was provided by the following shared resources at the H. Lee Moffitt Cancer Center: Biostatistics and Bioinformatics; Collaborative Data Services; and Participant Research, Interventions, and Measurement (PRISM).

## CONFLICT OF INTEREST

B.D.G.: Personal fees from SureMed Compliance and Elly Health, Inc. H.S.L.J.: Consultant with Red‐Hill Biopharma, Merck, and Janssen Scientific Affairs.

## ETHICAL STATEMENT

The study was approved by the Advarra Institutional Review Board. Data were collected as part of routine clinical care.

## AUTHOR CONTRIBUTIONS

*Conceptualization*, H.B., P.H.C., A.H., B.G., H.J.; *Methodology*, H.B., N.B., J.M.Z., B.G.; *Formal analysis*, H.B., N.B., J.M.Z.; *Resources*, P.J., H.J.; *Writing‐original draft*, H.B.; *Writing‐review & editing*, H.B., P.H.C., N.B., J.M.Z., A.H., B.G., P.J., H.J.; *Visualization*, H.B., N.B., J.M.Z.; *Supervision*, P.J., H.J.; *Funding acquisition*, H.J.; *Data curation*, H.B., P.H.C., N.B., J.M.Z., A.H.; *Validation*, N.B., J.M.Z.; *Project administration*, P.J.

## Supporting information


**TABLE S1** Multivariable logistic regression of Sum‐Max

**TABLE S2** Multivariable logistic regression of Severe‐Max

## Data Availability

The data that support the findings of this study are available from the corresponding author upon reasonable request.
