# Supplementary material for: Patient‐reported symptom burden in routine oncology care: Examining racial and ethnic disparities
Source: Cancer Rep (Hoboken). 2021 Jun 24;5(3):e1478. doi: 10.1002/cnr2.1478 (PMC8955049; doi:10.1002/cnr2.1478)
Supplement: Supplementary file 1 — TABLE S1 Multivariable logistic regression of Sum‐Max [file CNR2-5-e1478-s002.docx]

**Supplementary Table 1**. Multivariable Logistic Regression of Sum-Max

| **Variable** | **Level** | **Odds Ratio (95% CI)** | **OR P-value** | **Overall P-value** |
| --- | --- | --- | --- | --- |
| Gender | Female | 1.77 (1.23-2.55) | **0.002** | **0.002** |
|  | Male | - | - |  |
|  | | | | |
| Disease Status | No active disease | 0.64 (0.50-0.80) | **<.001** | **<.001** |
|  | Active disease | - | - |  |
|  | | | | |
| Cancer Site | Bone | 186911.4 (0.00-5.01E255) | 0.967 | **<.001** |
|  | Breast | 1.06 (0.60-1.86) | 0.844 |  |
|  | Endocrine | 5.03 (0.67-37.69) | 0.116 |  |
|  | GI | 2.92 (1.35-6.30) | **0.006** |  |
|  | GYN | 1.37 (0.60-3.13) | 0.451 |  |
|  | Genitourinary | 2.12 (0.80-5.62) | 0.131 |  |
|  | Head & Neck | 0.76 (0.48-1.22) | 0.255 |  |
|  | Hematologic | 2.16 (1.00-4.67) | 0.051 |  |
|  | Lung | 1.69 (1.02-2.81) | **0.043** |  |
|  | Male Genital | 0.61 (0.39-0.95) | **0.029** |  |
|  | Neuro | 1.24 (0.55-2.80) | 0.610 |  |
|  | Sarcoma | 1.28 (0.62-2.64) | 0.506 |  |
|  | Skin | - | - |  |

Note: Logistic regression model of the probability of having a nonzero total score. Cancer site was associated with a separation of data, evident in the infinite odds ratio estimate for cancer of the bone compared to cancer of the skin (reference). Thus, cancer site could not be included in the zero-inflation component of the ZINB model (Table 2b). The remaining variables, gender and disease status, were associated with the probability of having a nonzero total score and consequently carried forward into the ZINB model (Table 2b).
