# Supplementary material for: Patient‐reported symptom burden in routine oncology care: Examining racial and ethnic disparities
Source: Cancer Rep (Hoboken). 2021 Jun 24;5(3):e1478. doi: 10.1002/cnr2.1478 (PMC8955049; doi:10.1002/cnr2.1478)
Supplement: Supplementary file 2 — TABLE S2 Multivariable logistic regression of Severe‐Max [file CNR2-5-e1478-s001.docx]

**Supplementary Table 2**. Multivariable Logistic Regression of Severe-Max

| **Variable** | **Level** | **Odds Ratio (95% CI)** | **OR P-value** | **Overall**  **P-value** |
| --- | --- | --- | --- | --- |
| Age |  | 0.99 (0.98-1.00) | **<.001** | **<.001** |
|  | | | | |
| Gender | Female | 1.31 (1.10-1.55) | **0.002** | **0.002** |
|  | Male | - | - |  |
|  | | | | |
| Marital status: Married | Yes | 0.73 (0.64-0.84) | **<.001** | **<.001** |
|  | No | - | - |  |
|  | | | | |
| Disease Status | No active disease | 0.60 (0.52-0.68) | **<.001** | **<.001** |
|  | Active disease | - | - |  |
|  | | | | |
| Cancer Site | Bone | 2.53 (1.03-6.21) | **0.044** | **<.001** |
|  | Breast | 0.91 (0.67-1.22) | 0.517 |  |
|  | Endocrine | 1.85 (0.99-3.48) | 0.055 |  |
|  | GI | 2.05 (1.45-2.89) | **<.001** |  |
|  | GYN | 1.68 (1.11-2.53) | **0.013** |  |
|  | Genitourinary | 1.61 (1.00-2.60) | 0.052 |  |
|  | Head & Neck | 1.20 (0.89-1.62) | 0.225 |  |
|  | Hematologic | 1.83 (1.25-2.67) | **0.002** |  |
|  | Lung | 1.76 (1.32-2.34) | **<.001** |  |
|  | Male Genital | 0.61 (0.46-0.81) | **<.001** |  |
|  | Neuro | 1.20 (0.76-1.89) | 0.438 |  |
|  | Sarcoma | 1.67 (1.12-2.48) | **0.011** |  |
|  | Skin | - | - |  |

Note: Logistic regression model of the probability of having at least one severe symptom. Age, gender, marital status, disease status, and cancer site were associated with the probability of having at least one severe symptom and consequently carried forward into the ZINB model (Table 3b).
